# Stat3 Mediates Expression of Autotaxin in Breast Cancer

**DOI:** 10.1371/journal.pone.0027851

**Published:** 2011-11-28

**Authors:** Janeen Azare, Ashley Doane, Kenneth Leslie, Qing Chang, Marjan Berishaj, Jennifer Nnoli, Kevin Mark, Hikmat Al-Ahmadie, William Gerald, Maryam Hassimi, Agnes Viale, Mary Stracke, David Lyden, Jacqueline Bromberg

**Affiliations:** 1 Department of Medicine, Memorial Sloan Kettering Cancer Center, New York, New York, United States of America; 2 Department of Pathology, Memorial Sloan Kettering Cancer Center, New York, New York, United States of America; 3 Genomics Core Laboratory, Memorial Sloan Kettering Cancer Center, New York, New York, United States of America; 4 Department of Pediatrics, Memorial Sloan Kettering Cancer Center, New York, New York, United States of America; 5 Laboratory of Pathology, Division of Clinical Sciences, NCI, National Institutes of Health, Bethesda, Maryland, United States of America; 6 Department of Pediatrics, Weill Cornell Medical College, New York, New York, United States of America; Technische Universität München, Germany

## Abstract

We determined that signal transducer and activator of transcription 3 (Stat3) is tyrosine phosphorylated in 37% of primary breast tumors and 63% of paired metastatic axillary lymph nodes. Examination of the distribution of tyrosine phosphorylated (pStat3) in primary tumors revealed heterogenous expression within the tumor with the highest levels found in cells on the edge of tumors with relatively lower levels in the central portion of tumors. In order to determine Stat3 target genes that may be involved in migration and metastasis, we identified those genes that were differentially expressed in primary breast cancer samples as a function of pStat3 levels. In addition to known Stat3 transcriptional targets (*Twist*, *Snail*, *Tenascin-C* and *IL-8*), we identified *ENPP2* as a novel Stat3 regulated gene, which encodes autotaxin (ATX), a secreted lysophospholipase which mediates mammary tumorigenesis and cancer cell migration. A positive correlation between nuclear pStat3 and ATX was determined by immunohistochemical analysis of primary breast cancer samples and matched axillary lymph nodes and in several breast cancer derived cell lines. Inhibition of pStat3 or reducing Stat3 expression led to a decrease in ATX levels and cell migration. An association between Stat3 and the ATX promoter, which contains a number of putative Stat3 binding sites, was determined by chromatin immunoprecipitation. These observations suggest that activated Stat3 may regulate the migration of breast cancer cells through the regulation of ATX.

## Introduction

Breast cancer is the most common malignancy diagnosed among women worldwide [1]. Despite significant improvements in the diagnosis and treatment of this disease, tumor dormancy followed by distant recurrences accounts for 90% of all cancer deaths. Micrometastasis in the blood and bone marrow are the principal targets for adjuvant therapy [2], [3], [4], [5]. However, these metastatic cells can evade therapeutic interventions and eventually lead to recurrence. Clearly understanding the molecular mechanisms underlying the development of metastatic disease is required in order to treat this fatal disorder effectively.

Stat3 is a transcription factor which is known for its role as an integrator of cytokine and growth factor signaling [6]. Stat3 activation is dependent upon tyrosine phosphorylation, leading to dimerization between two Stat3 molecules. Activated Stat3 translocates to the nucleus where it binds to consensus promoter sequences of target genes and regulates their transcription. In contrast to normal cells where Stat3 activation is a transient process, Stat3 is persistently activated in a number of epithelial tumors including breast cancer and there is increasing evidence demonstrating that activated Stat3 plays a critical role in the pathogenesis of breast cancer including metastatic progression and response to therapy [7], [8], [9], [10], [11], [12], [13], [14], [15], [16], [17] [18], [19]. Breast tumors expressing high levels of activated Stat3 are inversely correlated with a complete pathological response to neo-adjuvant chemotherapy [20]. Inhibition of Stat3 activation in breast cancer cells inhibits growth and neo-angiogenesis, and potentiates a response to the chemotherapeutic agent doxorubicin [16], [21], [22]. Autocrine IL-6 production, a principal mediator of Stat3 activation in breast tumors, was found to be elevated in human mammary cancer/stem cells. Blockade of this signaling pathway reversed the aggressive features characteristic of basal-like breast cancers [23], [24]. In addition, side-population breast cancer stem-like cells express and require persistently activated Stat3 for viability and maintenance [25]. The mechanism(s) by which activated Stat3 mediates its effects is primarily through its ability to regulate gene transcription. Although a number of Stat3 target genes including vascular endothelial growth factor (VEGF), survivin, matrix metalloproteinase-9 (MMP-9) and twist have been identified in primary breast cancers and cancer-derived cell lines, we were interested in identifying additional target genes which may participate in metastatic progression of breast cancer [11], [20], [26], [27], [28], [29].

Autotaxin (ATX) or nucleotide pyrophosphatase-phosphodiesterase 2 (ENPP2), a secreted glycoprotein with lysophospholipase D activity, promotes cell migration, metastasis, and angiogenesis through the generation of lysophosphatidic acid (LPA), a lipid mitogen and motility factor that acts on several G protein-coupled receptors [30], [31], [32], [33], [34] [35]. Elevated levels of ATX have been demonstrated to play a role in migration and invasion of glioblastoma, lymphoma, hepatocellular carcinoma, melanoma and breast cancers, establishing this enzyme as a likely mediator of metastatic disease [36], [37], [38], [39], [40], [41], [42], [43], [44], [45], [46]. Significantly, enforced expression of ATX in metastatic models of breast cancer enhances osteolytic bone metastases while reduced expression of ATX inhibits bone metastases through regulation of osteoclasts [47]. Furthermore, it has recently been shown that over-expression of either ATX or LPA to the mammary gland mediates de novo tumorigenesis suggesting the oncogenic nature of this pathway [48], [49].

Here we examined primary breast cancer samples with matched axillary lymph nodes and observed a greater percentage of lymph nodes expressing moderate to high levels of pStat3 in contrast to the primary tumor. Upon further analysis of the primary tumors we determined that pStat3 levels were frequently highest along the leading edge of the primary tumor. These data suggested a role for pStat3 in mediating metastatic spread from the primary tumor to the axillary lymph nodes. By using gene expression profiling of primary breast tumors either expressing or lacking pStat3 protein, we identified a number of potential Stat3 target genes which may be involved in metastasis including *ENPP2 (ATX)*. The *ENPP2* promoter contains multiple Stat3 binding sites and an association between Stat3 and these binding sites was observed. We demonstrated a positive correlation between moderate to high levels of pStat3 and ATX in primary breast tumors and lymph nodes as well as in several breast cancer derived cell lines. Activated Stat3 mediated ATX up-regulation and enhanced migration of breast cancer cell lines. Conversely, inhibition of Stat3 activity blocked migration with a concomitant decrease in ATX levels. In summary, we have identified ATX as a putative novel Stat3 target gene in breast cancer.

## Materials and Methods

### Samples and Gene Expression Analysis

Tissue samples were obtained from therapeutic or diagnostic procedures performed as part of routine clinical management at Memorial Sloan-Kettering Cancer Center (MSKCC). All research procedures using human tissue were approved by the MSKCC institutional review board (06-177). Patients signed an informed consent demonstrating their interest and willingness to have their tissues analyzed for research purposes. Tissues were snap frozen in liquid nitrogen and stored at −80°C. Each sample was examined histologically using hemotoxylin and eosin-stained cryostat sections and enriched for areas of interest by manual trimming of tissue blocks. Isolation of RNA, cDNA synthesis and gene expression analysis using the HG-U133A microarray was previously described [50]. The expression data on these tumor samples can be accessed using http://caarraydb.nci.nih.gov/caarray/. We used samples which were definitively pStat3+ versus pStat3 negative throughout all cell types within the tumor for our gene expression analysis. Affymetrix.CEL files were analyzed using tools in Partek Genomic Suite 6.4 software (Partek Inc). The Robust Multi-array Analysis (RMA) algorithm was used for global normalization and probeset summarization. To identify differentially expressed genes between two groups (pStat3+ versus pStat3−), a students T-test was performed followed by a 2-fold change filter. The statistically significant genes (214 gene list of differentially expressed genes) were used to perform two-way hierarchical clustering of tumor samples. Pearson dissimilarity metric and average linkage method were used to calculate the dendrogram.

### TMAs and immunohistochemistry

Multi-tissue blocks of formalin-fixed, paraffin- embedded primary breast cancer and matched axillary lymph nodes (containing 3 or 4 representative 0.6-mm cores) were prepared using a tissue arrayer, and immunohistochemistry was performed as described previously [23], [50]. Antigen retrieval, with the use of citric acid (pH 6.0) at 97°C for 30 minutes, was followed by treatment with 3% hydrogen peroxide. pSTAT3 (Tyr705) (Cell Signaling; #9131) and ATX (M. Stracke) were used at 1∶200 and 1∶1000 respectively [23], [43]. The ATX rabbit polyclonal antibody was affinity purified and generated against a C-terminal peptide which allows for recognition of all 3 isoforms of ATX as well as both cytosolic and secreted forms of ATX [51], [52], [53], [54]. Denatured recombinant ATX was used as a blocking agent for IHC and these controls revealed no staining demonstrating specificity of this antibody for IHC (data not shown). Secondary reagents were from DakoCytomation EnVision+ Dual Link System-HRP (DAB+) kit or the DakoCytomation LSAB+ system-HRP kit. Counterstaining was performed with the use of hematoxylin. Scoring of the TMAs was performed by 2 independent observers, with a high correlation observed between scorers (*P*<0.001) for both pSTAT3 and ATX. For a tumor to be considered positive for either pSTAT3 or ATX, all 3–4 replicates in the tissue array had to have a similar staining intensity, otherwise, it was excluded. pStat3 levels were subjectively graded as a function of relative nuclear staining intensity: No/low staining of epithelial cells (0–1+) and moderate/high staining (2–3+). We also classified pStat3 levels within the stromal and immune compartment of the tumor and for our gene expression analysis we excluded those samples which were positive for pStat3 in the tumor microenvironment yet negative in the epithelial compartment. We classified tumors as expressing no cytoplasmic ATX (0), low staining (+1) and moderate/high staining (+2–3). Statistical analyses were done using StatView (SAS Institute). The correlation between the scores of both scorers and the relation between those of pSTAT3 and ATX were measured by using the χ2 test.

### Cell culture and Reagents

Human breast cancer MDA-MB-435, MDA-MB-231, BT474, MCF-7, 1937, 1806, 1143, 38, 1833 and 4175 cells (ATCC and kindly provided by J. Massague [55], [56], [57] were cultured in DME HG: F12 P+S+NEAA containing 10% fetal bovine serum (Invitrogen). 1833Stat3Sh and 1833CSh cells were generated using a Stat3Sh lentiviral construct as well as a scrambled control construct [58]. Cells were treated with 5 ng/ml oncostatinM (Chemicon) or 1 µM P6 pan-Jak inhibitor (Calbiochem). ATX rabbit polyclonal antibody was used at 1∶10,000 for western blot analysis and 1∶1000 for IHC (M. Stracke-see description above for details of antibody) [51], [52], [53], [54]. pStat3 rabbit polyclonal antibody (Cell Signaling, #9131) was used at 1∶1000 for western blot and 1∶200 for IHC. Stat3 rabbit polyclonal antibody (Cell Signaling, #9132) was used at 1∶1000 for western blotting. Anti-Stat3 antibody for ChIP was used at 1∶200 (Santa Cruz, #C-20X). Tubulin monoclonal antibody (Sigma) was used at 1∶10,000. Vimentin antibody for western blot was used at 1∶1000 (Sigma). pStat5 antibody for western blot was used at 1∶1000 (Cell Signaling).

### Cell lysate preparation and Western blotting

Cells were treated with or without 5 ng/ml oncostatinM (OSM; Chemicon International, Inc.) for 1 h or with or without 1 µM Pyridone 6 (Calbiochem) for 24 h, and then whole-cell lysis extracts were obtained: 150 mM NaCl, 10% glycerol,1% IGEPAL, 0.5% sodium deoxycholate, 2 mM EDTA, 1 mM NaF, 1 mM Na3VO4, 1 mM Na2MoO4, 0.1% SDS, 20 mM Tris-HCl, pH 8 with proteinase inhibitors. Western blots analysis was performed as previously described [26].

### Reverse-transcriptase-PCR (RT-PCR) amplification

Total RNA was isolated from cells treated with or without OSM for 4 h with or without 1 mM P6 for 24 hr using the RNeasy© Mini Kit (Qiagen Inc.) according to the manufacturer's instructions followed by reverse transcription. The cDNA samples were amplified by PCR using 32P-labeled deoxynucleosidetriphosphate. Primers for ATX were: sense, 5′-CGT GAA GGC AAA GAG AAC ACG-3′, and antisense, 5′-AAA AGT GGC ATC AAA TAC AGG-3′, of the ATX cDNA, producing a 784 bp product. Primers for the β-actin internal control housekeeping gene were: sense, 5′-CGT GCG TGA CAT TAA GGA GA-3′, and antisense, 5′-TGA TCC ACA TCT GCT GGA AG-3′, producing a 450 bp product.

### Chromatin immunoprecipitation

Chromatin immunoprecipitation assays were done on MDA-MB-435, 1833CSh and 1833S3Sh cells either treated with DMSO control, OSM for 30 minutes or P6 for 4 hrs.using a chromatin immunoprecipitation assay kit (Upstate Biotechnology). Stat3-DNA complexes were precipitated by using anti-Stat3 antibody (#C-20X, Santa Cruz). Polyclonal IgG antibody was used as a negative control. Precipitated DNA was amplified by PCR using primers flanking the gamma-activated sites (GAS) sites at −821 - 5′-CGAAACAAGCTGACAG and −301 - 5′-GGCCCATAACAGTGCATGTTC A 58°C annealing temperature using 30 cycles was used for amplification.

### Cell migration assay (Boyden chambers)

MDA-MB-435 and 1833 derived cell lines were preincubated for 16 h in 1% serum-containing medium and conditioned media (CM) was collected from these. Migration assay was performed using a 24-well cell culture plate (Becton Dickinson) with 8.0-µm pore membrane inserts (Becton Dickinson). To the lower chambers, conditioned media from MDA-MB-435 and or 1833 derived cell lines either treated for 16 hr with OSM (5 ng/ml) or P6 (1 µM) was added to the lower chamber. MDA-MB-435 and 1833 derived cell lines (1×10^5^) were added to the upper wells, and the chambers were incubated for 24 h at 37°C. Migrated cells were visualized with 0.5% crystal violet in 20% methanol and counted. Each condition was assayed in triplicate, experiments were performed independently at least three times, and the results were expressed as the number of cells per field. A one-way analysis of variance was used to determine significance.

## Results

### Tyrosine phosphorylated Stat3 in breast cancer and matched metastatic axillary lymph nodes

**Figure 1:**
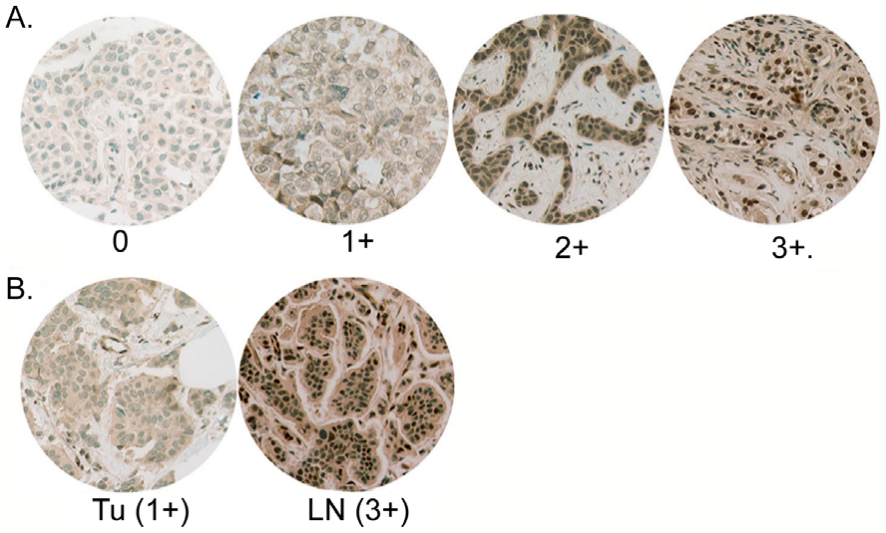
Tyrosine phosphorylated Stat3 in breast cancer and matched metastatic axillary lymph nodes. **A**. Tissue microarrays (TMAs) of primary breast tumors (38) were analyzed for nuclear tyrosine-phosphorylated Stat3 (pStat3) by immunohistochemical (IHC) analysis. Examples of no detectable pStat3 (0), low levels (1+), moderate levels (2+) and high levels (3+) are shown. **B**. Comparison of pStat3 levels of tumor cells by IHC in matched primary tumors with axillary lymph nodes. 37% of primary tumors expressed 2–3+ pStat3, while 63% of matched lymph nodes expressed 2–3+ pStat3. Relative pStat3 IHC staining was greater in the lymph node compared to the corresponding primary tumor in 15/38 specimens, equal in 18/38 and less in 5/38. A representative example of a tumor (Tu) specimen with 1+ pStat3 versus 3+ pStat3 in the corresponding lymph node (Ln) is shown.

**Figure 2:**
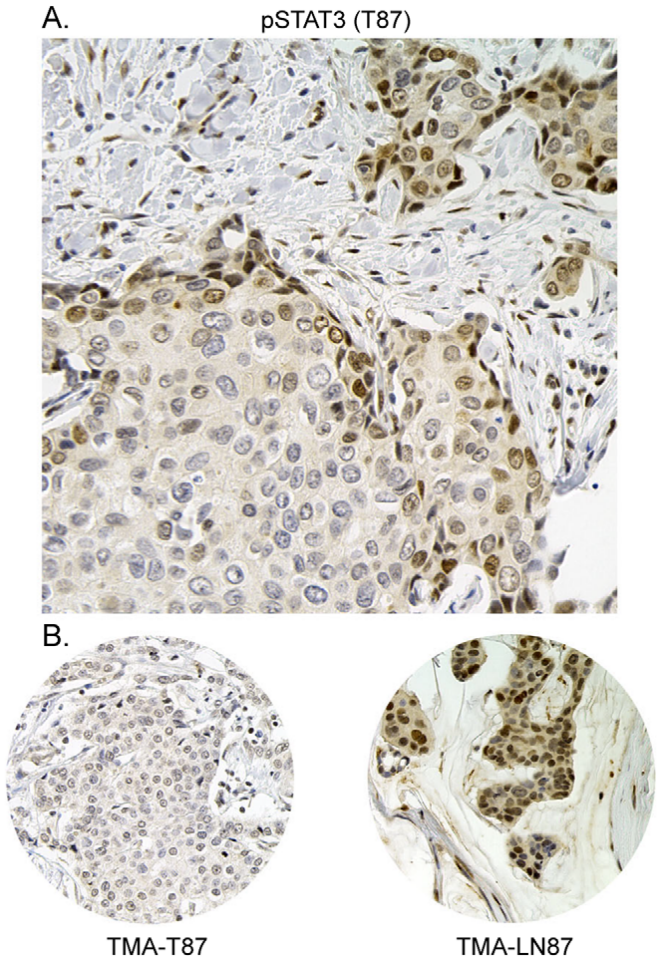
Tyrosine phosphorylated Stat3 levels are greater along the leading edge of tumors. IHC for pStat3 was performed on the primary tumor specimens (38) from which the cores for the TMA were derived from. **A.** A representative example demonstrating relatively high levels of pStat3 (2+) along the leading edge of the tumor. **B.** IHC for pStat3 in the corresponding tissue microarray 0.6 mm core for both the tumor and matched axillary lymph node.

We have described the prevalence of moderate to high levels of tyrosine phosphorylated Stat3 (pStat3) in primary breast cancers to be 46% with no association with estrogen, progesterone or Her2/neu receptor expression [23]. These findings are consistent with other reports [9], [20], [59], [60], [61]. Furthermore, high levels of pStat3 within primary tumors are associated with metastasis to regional lymph nodes [28]. However, the relationship between pStat3 in primary tumors and matched axillary lymph node metastases has not been described. We examined tissue micro-arrays (TMAs) of 38 primary breast tumors with matched axillary lymph node metastases for the relative expression of pStat3 by immunohistochemistry. pStat3 levels were subjectively graded as a function of relative nuclear staining intensity: No/low staining of epithelial cells (0–1+) and moderate/high staining (2–3+) defined negative and positive pStat3 expression, respectively. 14/38 (37%) of primary tumor samples were IHC positive (2–3+); while 24/38 (63%) of matched lymph node metastases were IHC postive (2–3+) (Fig. 1A and B). We also observed that the relative pStat3 IHC staining was greater in the lymph node metastasis compared to the corresponding primary tumor in 15/38 specimens, equal in 18/38 and less in 5/38 (Fig. 1B). Given that tumors are heterogeneous, we hypothesized that a subset of breast cancer cells expressing activated Stat3 were capable of metastasizing to the axillary lymph nodes. To this end, we examined pStat3 levels and distribution of whole tumor sections from the original paraffin tissue blocks from which the 1 mm cores were obtained to generate the TMAs. We determined that pStat3 levels were highest along the edge of the tumor section in >90% of tumors while pStat3 staining within the lymph node metastases was more uniform in distribution (Fig. 2). This finding was not however due to an artifact of antigen retrieval as areas of normal breast surrounding the tumor section were negative for pStat3 (data not shown).

### Identification of Stat3 regulated genes involved in metastasis

In order to identify potential Stat3 target genes in breast cancer which may regulate invasion, migration and metastasis we sought to identify genes that were differentially expressed in tumor samples as a function of pStat3 as assessed by IHC. Gene expression profiling had been performed on 99 primary breast cancer samples (42 ER− and 57 ER+) and we previously reported that 46% of these were immunohistochemically positive for pStat3 [23], [50]. Those samples from the TMA which expressed moderate to high levels of pStat3(2–3+) in tumor epithelial cells versus those that were negative for pStat3 were further evaluated by examining pStat3 levels and tissue distribution from sections of the original paraffin blocks containing the tumor specimens. Many of the specimens that were negative for pStat3 in the tumor expressed moderate to high pStat3 levels in surrounding lymphocytes, endothelial cells or stromal cells (data not shown). However, we identified 16 specimens which expressed no pStat3 staining in tumor cells, lymphocytes, endothelial cells or stromal cells. 13/16 of these pStat3 negative specimens were ER negative. Given the significant effect that ER has on gene expression and the potential for molecular cross talk between Stat3 and ER signaling, we chose to restrict our initial analysis to the ER− subset of tumors in order to identify genes associated with Stat3 activation.

**Figure 3:**
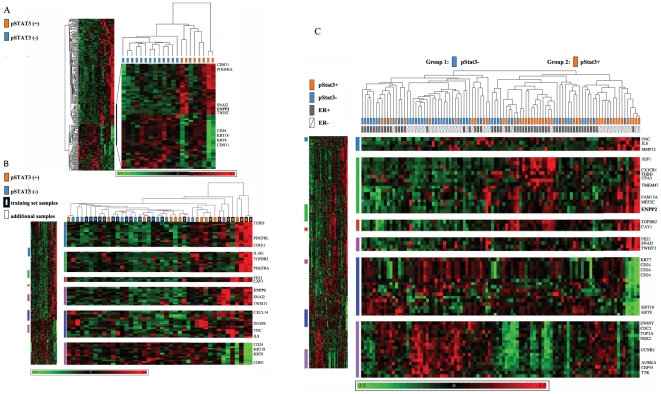
Hierarchical Cluster Analysis by pStat3. **A.** Two-way hierarchical clustering was performed with the 21 ER− pStat3 (+/−) samples using the gene expression values limited to the 214 differentially expressed genes. The region of the dendrogram including ENPP2 is enlarged. **B.** The same analysis as described in A was performed with the 41 ER− samples. Several regions of the dendrogram including ENPP2 were enlarged **C.** Two-way hierarchical clustering was performed with all (98) of the tumor specimens categorized by pStat3 status (+/−) using the 214 differentially expressed genes. The dendogram represents the relationship among samples, and the length of branches represents 1 minus the pearson correlation coefficient between two samples. Group 1 are primarily pStat3 negative and Group 2 are pStat3 positive. Samples are arranged in columns and genes in rows. Normalized expression levels are pseudocolored red to indicate transcript levels above the median for that gene across samples, and green for expression below the median. Color saturation is proportional to the magnitude of expression. Several clusters of differentially expressed genes including ENPP2 are enlarged. ER and pStat3 status are indicated for each sample (grey versus white, orange versus blue).

Of the 42 ER− tumor samples, 8 were strongly positive for pStat3 (3+ in tumor cells only) and 11 were moderately to strongly positive for pStat3 (2–3+ in the tumor) but also had many infiltrating pStat3+ stromal, endothelial cells and/or lymphocytes. In contrast, 13 were negative for pStat3 (in tumor and surrounding stromal, endothelial cells and lymphocytes), 5 were pStat3− in the tumor cells but pStat3+ in the infiltrating lymphocytes, endothelial or stromal cells and 5 were weakly pStat3+ (0–1+) within the tumor cells. Given that our samples had not undergone laser capture dissection we felt it necessary to distinguish those samples “contaminated” with pStat3+ stromal and immune cells in our gene expression analysis. We were interested in identifying those genes which were differentially expressed predominantly as a function of the expression of pStat3 within the tumor/epithelial compartment. A microarray statistical analysis of the 8 pStat3+ (2–3+ in tumor cells only) versus the 13 pStat3− (0 in tumor, endothelial, stromal and lymphocytes) tumor specimens led to the identification of 214 genes which were differentially expressed according to pStat3 status (at least two-fold between the means of pStat3(+) and pStat3(−) cases and a Student's *t*-test *P*<0.05). Of the 214 differentially expressed genes, 150 genes were over-expressed and 64 genes were under-expressed in pStat3(+) cases relative to pStat3(−) cases (Table S1). Differentially expressed genes included several previously identified Stat3 targets such as *Twist*, *Snail* and *IL-8* [29], [62], [63], [64], [65], [66], [67], [68], and this suggested our analysis at least partially captured a molecular profile of Stat3 transcriptional activation in breast cancer. Interestingly, the top ranked differentially expressed gene according to significance testing (p<0.0001) and fold change (8.6 fold) was *ENPP2* which encodes ATX, a secreted glycoprotein that promotes cell migration, metastasis, and angiogenesis. Two-way hierarchical clustering was performed using expression of the 214 differentially expressed genes in 21 ER(−) breast cancers with definitive pStat3+(orange rectangles) or pStat3−(blue rectangles) status. This analysis demonstrated a clear separation of these two groups as expected. The cluster of genes containing ENPP2 is magnified to illustrate its expression pattern across the samples (Fig. 3A). We also included the remaining 21 ER− tumor specimens in our clustering analysis which either expressed low levels of pStat3 (0–1+) within tumor cells with variable levels of pStat3 within the “microenvironment” (blue rectangles without borders) versus moderate to high levels of pStat3 (2–3+) within tumor cells also with variable levels of pStat3 in the microenvironment (orange rectangles without borders) which also revealed good separation of tumor specimens as a function of pStat3 status as expected(Fig. 3B). In addition, hierarchical clustering on all of the tumor specimens for which pStat3 status had been determined (42 ER− and 57 ER+) revealed two sample groups associated with pStat3 status (Fig. 3C). Within the pStat3 positive group (group 2) ENPP2/ATX levels appeared to be elevated in both the ER+ (grey rectangles) and the ER− (clear rectangles) specimens relative to the pStat3 negative group. To further explore this expression pattern we repeated the statistical analysis using all of the tumor specimens to identify differentially expressed genes as a function of pStat3 (blue versus orange samples) and 136 differentially expressed genes were identified (Table S2). A comparison of this gene list with the 214 pStat3 gene list revealed 50 genes overlapping including *ENPP2* (Table S3) as expected. Given the known significance of ENPP2/ATX as a key regulator of tumor cell migration, these data suggested a role for ATX in regulating the metastatic potential of pStat3(+) breast cancers [33], [38], [42], [43] [47], [49].

### ATX expression in breast cancer correlates with pStat3

**Figure 4:**
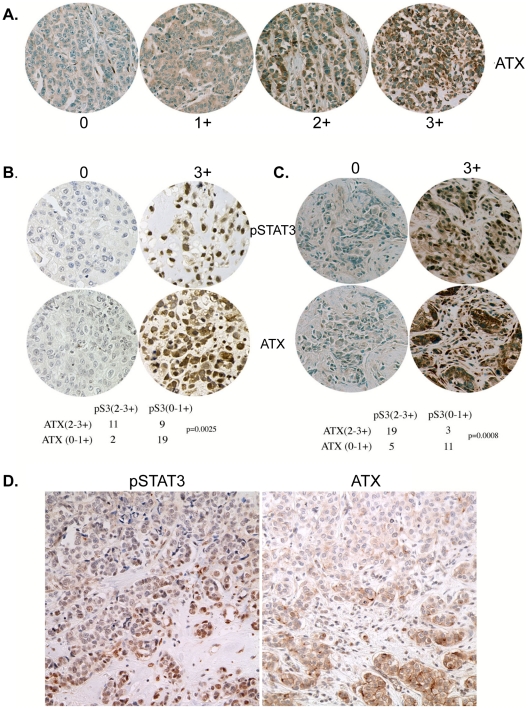
ATX in primary breast tumors and matched axillary lymph nodes positively correlate with pStat3. **A.** TMAs of 41 ER− breast tumors were analyzed for cytoplasmic ATX levels by IHC analysis. Representative tumors of no detectable ATX (0), low levels (+1), moderate levels (+2) and high levels (+3) are shown. 49% expressed moderate to high levels of ATX (2–3+) whereas 51% expressed low levels of ATX. **B.** A positive correlation between (2–3+) pStat3 and (2–3+) ATX levels was observed in the primary tumors (p = 0.0025). Examples of both low (0) and high (3+) ATX with corresponding serial sections for pStat3 are shown. **C.** A positive correlation between (2–3+) pStat3 and (2–3+) ATX levels was observed in the 38 lymph nodes described in Fig. 1 (p = 0.0008). Examples of both low (0) and high (3+) ATX with corresponding serial sections for pStat3 are shown. **D.** A representative example of serial sections of a tumor expressing moderate pStat3 and ATX levels on the tumor edge.

By immunohistochemical analysis we next examined the levels of ATX in the 42 ER− primary breast tumors. Tumors were classified as expressing no ATX (0; 24%), low staining (1+; 27%) and moderate/high staining (2–3+; 49%) (Fig. 4A). A positive correlation between moderate/highATX and pStat3 levels (2–3+) was determined by x^2^ analysis (Fig. 4B; p = 0.0025). The 38 primary tumors with matched axillary lymph nodes were also examined for ATX levels. 12/38 (32%) primary tumors expressed moderate to high (2–3+) levels of ATX; while 22/38 (58%) lymph nodes were (+2–3) positive for ATX (data not shown). Furthermore, a positive correlation between ATX and pStat3 expression within lymph nodes was observed p = 0.0008 (Fig. 4C). We also examined the distribution of ATX levels in the whole tumor section as was performed for pStat3 in Fig. 2. Here we also observed relatively higher levels of ATX on the edge of the tumor specimens (Fig. 4D).

### Stat3 regulates ATX expression and cell migration in breast cancer cells

**Figure 5:**
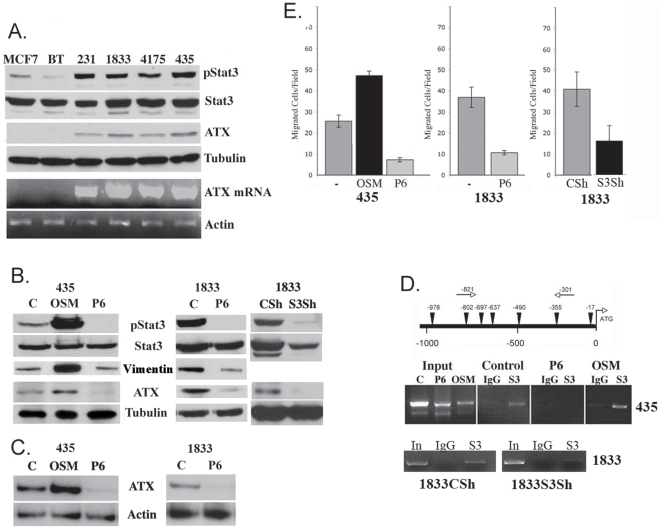
Stat3 regulates ATX expression and cell migration in breast cancer cells. **A**. Extracts (20 µg) isolated from MCF7, BT474 (BT), MDA-MB-231 (231), 1833, 4175 and MDA-MB-435 (435) were analyzed for pStat3, Stat3, ATX and Tubulin by western blot analysis. Total RNA was isolated from the same cell lines and analyzed for *ATX* by RT/PCR and normalized to Actin. **B**. Whole cell extracts (50 µg) isolated from MDA-MB-435 cells treated for 4 hours with dimethyl sulfoxide (C), OSM (5 ng/ml) and P6 (1 µM); 1833 cells treated with DMSO or P6 and 1833 Control Sh (CSh) or expressing a Stat3 Sh (S3Sh) were analyzed for pStat3, Stat3, Vimentin, ATX and Tubulin by western blot analysis. **C**. Total RNA isolated from MDA-MB-435 cells treated for 4 hours with dimethyl sulfoxide C, OSM and P6; and 1833 cells treated with DMSO (C) or P6 was analyzed for ATX by RT/PCR and normalized to Actin. **D**. Potential (TTN5AA) Stat3 binding sites are indicated by inverted triangles on the ATX promoter. Arrows indicate the direction of transcription and nucleotide positions are shown below the diagram. MDA-MB-435 cells treated for 30 minutes with dimethyl sulfoxide (C), OSM (5 ng/ml) and P6 (1 µM) were subjected to chromatin immunoprecipitation assay using antibodies to to Stat3 (S3) or IgG as a negative control. Similarly, 1833 Control Sh (1833CSh) or 1833 cells expressing a Stat3Sh (1833S3Sh) were were subjected to chromatin immunoprecipitation assay using antibodies to Stat3 (S3) or IgG as a negative control. Co-precipitated DNA was amplified by PCR using primers flanking the Stat3 binding sites at −821 and −301. The input was 5% of the total. **E**. Migration of MDA-MB-435 cells in a Boyden chamber assay was determined in the presence of conditioned media from dimethyl sulfoxide (-), OSM (5 ng/ml) or P6 (1 µM) treated MDA-MB-435 cells. Migration of 1833 cells was determined in a Boyden chamber assay in the presence of conditioned media from 1833 cells from dimethyl sulfoxide (-) and P6 treated 1833 cells. Migration of 1833 Control Sh (CSh) and 1833 cells expressing a Stat3Sh (S3Sh) was determined in a Boyden chamber assay with conditioned media in the bottom chamber from the same corresponding cell lines. Results are expressed as the number of cells per field (as determined by crystal violet staining) and are the mean SD of triplicate values from three independent experiments.

In order to characterize the relationship between activated Stat3 and ATX, we examined a number of breast cancer derived cell lines for expression of ATX message and protein relative to pStat3 (Fig. 5A, 5B and 5C). MCF-7 and BT474 breast cancer derived cell lines expressed low levels of pStat3 and no to low expression of ATX mRNA; while 1937, 1806, 1143, 38, MDA-MB-231, 1833, 4175 and MDA-MB-435 cells expressed high levels of pStat3 protein and ATX mRNA (Fig. S1A and Fig. 5A). MDA-MB-435, 231, 4175 and 1833 cells express activated Stat3 as a consequence of persistant activation of gp130 signaling [23]. Activated Stat5 has been reported to regulate some of the same genes as activated Stat3 [18], [19]. We determined that 1833 and to a lesser degree 4175 cells expressed pStat5, while MCF-7, BT474, MDA-MB-231 and MDA-MB-435 cells did not (Fig. S1B and S1C). The role of pStat5 in the regulation of ATX is not clear. Jak inhibition (P6) reduced both pStat3 and ATX but had no effect on pStat5 levels in 1833 cells, suggesting a marginal role for this transcription factor in ATX regulation.

Stimulation of MDA-MB-435 cells with oncostatin M (OSM), a growth factor which activates the gp130/Jak/Stat3 pathway, led to an increase in the levels of activated Stat3 with a concomitant increase in ATX protein and mRNA levels (Fig. 5B, 5C). In corcordance with this finding, inhibition of gp130 signaling using a pan-Jak inhibitor (P6) led to a marked decrease in pStat3 and ATX levels in all of the described cell lines (Fig. 5B, 5C and data not shown), suggesting at Stat3 dependent mechanism. Vimentin (a marker of EMT) levels also increased in response to OSM and were reduced in response to P6 (Fig. 5B). In order to demonstrate that Stat3 rather than pan-Jak inhibition was responsible for mediating ATX expression, we knocked-down Stat3 levels using a Stat3 ShRNA (S3Sh) in 1833 cells and determined that ATX protein and RNA (data not shown) levels were decreased compared to cells infected with a scrambled control ShRNA (CSh) (Fig. 5B) [58], [69].

To investigate the role of Stat3 in the transcriptional regulation of the human *ATX* gene, we examined 1000-bp of the *ATX* promoter and identified 7 putative Stat3 binding sites (TTN5AA; N≥2GC residues) (Fig. 5D). Cross-linking chromatin immunoprecipitation assays were carried out to determine whether the potential Stat3 binding sites within the *ATX* promoter bound activated Stat3. Primer sets were used to target the regions of the *ATX* promoter corresponding to the Stat3 binding sites found between −821 and −301. Cell extracts from MDA-MB-435 cells were subjected to chromatin immunoprecipitation (ChIP) analysis with antibodies to Stat3 or IgG (negative control). Amplification of the *ATX* promoter by PCR was observed in the samples expressing activated Stat3 (Control and OSM stimulated) but not in the P6 treated samples (Fig. 5D). Similarly, ChIP analysis of Stat3 bound to the *ATX* promoter was performed on 1833 cells expressing activated Stat3 (1833CSh) compared to those expressing less Stat3 (1833S3Sh) revealing PCR amplification of the ATX promoter in the Stat3 expressing line (1833CSh) (Fig. 5D). These data demonstrate that activated Stat3 can associate with the human *ATX* promoter.

ATX is a known mediator of cell migration, invasion and angiogenesis principally through the production of LPA [70]. Here we examined the effect of increasing or decreasing pStat3/ATX levels on MDA-MB-435 and 1833 cell migration. Migration was measured using a Boyden chamber assay in the presence of conditioned media (CM) from MDA-MB-435 or 1833 cells untreated, stimulated with OSM or treated with P6. We observed enhanced migration of cells in the presence of CM from OSM treated cells compared to control CM from MDA-MB-435 cells and inhibition of migration with CM from P6 treated MDA-MB-435 and/or 1833 cells (Fig. 5E). We also demonstrated reduced cell migration in the presence of CM from 1833 cells lacking Stat3 (1833S3Sh) compared to CM from control 1833 cells (Fig. 5E). Taken together, these data suggest that activated Stat3 upregulates ATX expression which leads to an increase in cell migration. Conversely, inhibition of Stat3 leads to a reduction in the expression of ATX and a corresponding decrease in cell motility.

## Discussion

Stat3 is persistently tyrosine phosphorylated in a large number of tumors of epithelial origin including breast cancer. However, its precise role in breast tumorigenesis has been an area of significant investigation. Examination of the distribution and incidence of pStat3 levels by immunohistochemical analysis and correlating these data with prognostic features has led to seemingly contradictory results. Specifically, elevated levels of phosphorylated Stat3 in tumor samples from 45 patients with high-risk breast cancer was correlated with an incomplete response to neo-adjuvant chemotherapy (i.e. poor prognostic feature) [20]. However, analysis of tissue microarrays of 346 node-negative patients revealed that postive phosphorylated Stat3 levels was a prognostic marker of improved overall survival [61]. A number of reasons could account for this apparent discrepancy including: 1. The stage of the disease StageIII [20] versus StageI [61] 2. Differences in pStat3 levels in fresh samples [20] versus archived samples >1–25 years old [61]. 3. The presence of pStat5, which has been shown to be associated with more differentiated breast tumors and with a more favorable prognosis. Furthermore, pStat5 can exert a dominant role over Stat3 [19]. 4. The analysis of whole tumor blocks [20] versus 1mm cores [61].

Our analysis of pStat3 levels of primary tumor sections initially from tissue microarrays (TMAs) and subsequently from whole tumor blocks revealed that 20% of those which were initially scored as low for pStat3 (analysis of the TMA) were in fact positive along the edge or rim of the tumor adjacent to stromal cells when the corresponding tumor block was analyzed (Fig. 2). Furthermore, the highest pStat3 levels were almost always found on the edge of tumor samples. Similar observations have been made in non-small cell lung cancer samples where pStat3 levels were concentrated in tumor cells adjacent to non-tumor stromal tissues [71]. This study is the first to examine the relative levels of pStat3 in primary breast tumors with matched axillary lymph nodes. Here we show that a greater fraction of lymph nodes express moderate/high levels of pStat3 compared to the corresponding primary tumors (Fig. 1B). One explanation for this observation is that cytokines such as IL-6 which mediate Stat3 activation in breast cancers are expressed at higher levels in lymphatic tissue resulting in increased pStat3. However, we did not observe high IL-6 levels within lymphocytes in the affected lymph nodes, rather high IL-6 levels were observed within the tumor epithelial cells which correlated with pStat3 levels (manuscript submitted, JFB) [23]. An alternative explanation for the relative increase in pStat3 staining in lymph nodes versus primary tumor is that the pStat3 positive cells within primary breast tumors have an increased migratory and invasive capacity compared to the pStat3 negative expressing cells. Indeed it has been demonstrated that tumors and tumor-derived cell lines are heterogeneous in nature leading to the development of sub-clones with differential capacity to metastasize to various organs [55], [56], [57], [72].

Stat3 has increasingly been shown to play a role in migration and invasion of both normal and cancer cells. Stat3 through LIV1/Snail/Twist controls epithelial-mesenchymal transition in zebrafish gastrula organizer [66], [73]. Activated Stat3 is a direct transcriptional regulator of Twist in breast cancer which is a critical regulator of metastatic progression [16], [29], [74], [75]. Conditional deletion of Stat3 in the skin has revealed a requirement for it in wound healing and tumorigenesis [76], [77]. Stat3 knockdown in murine models of ErbB2-mediated breast carcinoma altered epithelial adhesion and polarity [17]. Stat3 in conjunction with NF-kB is a transcriptional regulator of the chemokine RANTES which was recently shown to be a potent mediator of metastatic spread of breast cancer cells [78], [79]. We have shown that Stat3 regulates expression of MMP-9 in breast cancer and integrin ß6 in prostate cancer which are critical regulators of invasion and migration [26], [80]. In order to identify additional Stat3 targets responsible for metastatic spread, we used gene expression profiling of primary breast tumors [50]. We compared the gene expression profiles of samples expressing moderate/high levels of pStat3 within carcinoma cells to those with no activated Stat3 in either carcinoma cells, lymphocytes, stromal cells and endothelial cells. In addition to the *ENPP2/ATX* gene, a number of previously identified Stat3 targets were identified using this approach including including *Twist*, *Snail*, *Tenascin-C*, *IL-8* and *E-Cadherin* [29], [62], [63], [64], [65], [66], [67], [68], [69], [80]. We have observed an inverse correlation between pStat3 and E-Cadherin in primary breast tumors (manuscript under review, JFB). Phoshorylated Stat3 and Vimentin (a marker of EMT) increased in 435 cells treated with OSM, while P6 treatment of 1833 cells led to a reduction of both pStat3 and Vimentin (Fig. 5B). It is interesting that genes over-expressed in triple negative breast cancers and in highly metastatic cancer derived cell lines express a number of these same transcripts [81], [82].

ATX or ENPP-2 is a secreted lysophospholipase which mediates production of LPA, a stimulator of breast cancer migration and whose expression is associated with mammary tumorigenesis, breast cancer metastasis, angiogenesis and survival [31], [38], [43], [49], [83]. MDA-MB-435 cells express moderate levels of ATX and pStat3, yet its expression is markedly increased following the addition of a gp130 ligand leading to increased pStat3 levels, which resulted in an increased chemotactic potential (Fig. 5). There has been some controversy about the origin of MDA-MB-435 cells with some studies suggesting it may be of melanoma origin [84]. However, a recent study suggests that the manner in which MDA-MB-435 cells are propogated can lead to differential expression of epithelial and breast specific markers as well as melanocyte-specific markers suggesting lineage infidelity [85], [86]. In light of this controversy we expanded our cell line analysis to others (Fig. 5). It was recently reported that NFAT1 could transcriptionally regulate the ATX gene in integrin α6ß4 transfected MDA-MB-435 cells [33]. There is an NFAT1 binding site at −327 (27 bp away from a Stat3 binding site). However, no known synergistic associations between these two transcription factors have been described. In addition, v-Jun expression in chick embryo fibroblasts led to an increase in ATX mRNA levels, though a direct transcriptional mechanism was not determined [87]. Stat3 has been shown to interact with the AP-1 proteins c-jun and c-fos, however no ideal AP-1 binding sites were identified within the ATX promoter region [88]. Although our data demonstrates a direct interaction between Stat3 and the ATX promoter, other Stat3 targets or upregulated transcripts (VEGF, NRP1 and CD36) may be participating in the transcriptional regulation of *ATX* [54], [89]. In summary we have determined that activated Stat3 expression is heterogeneous in distribution in primary breast tumors with the highest levels found on the tumor edge. Furthermore, a relative increase in pStat3 staining in matched axillary lymph nodes versus primary tumor suggesting that pStat3 positive cells have an increased capacity to metastasize to lymph nodes. Finally, using gene expression profiling of primary tumors, we identified *ENPP2/ATX* as a novel Stat3 target gene involved in cell migration and invasion.

## Supporting Information

Figure S1 **Stat3 regulation of ATX expression in triple negative breast cancer cells.** **A.** Extracts (20 µg) isolated from 1937, 1806, 1143, 38 and MD-MB-231 cell lineswere analyzed for pStat3, ATX and Actin by western blot analysis. **B.** Extracts (20 µg) isolated from MCF7, BT474 (BT), MDA-MB-231 (231), 1833, 4175 and MDA-MB-435 (435) were analyzed for pStat3, Stat3, pStat5, ATX and Tubulin by western blot analysis. **C.** Whole cell extracts (50 (µg) isolated from MDA-MB-435 cells treated for 4 hours with dimethyl sulfoxide (C), OSM (5 ng/ml) and P6 (1 µM); 1833 cells treated with DMSO or P6 were analyzed for pStat3, Stat3, Vimentin, pStat5, Stat5, ATX and Tubulin by western blot analysis.
(TIF)

Table S1 **Differential gene expression in primary ER− breast cancer as a function of pStat3.** A microarray statistical analysis of the 8 pStat3+ versus the 13 pStat3− tumor specimens identified 214 differentially expressed genes according to pStat3 status (at least two-fold between the means of pStat3(+) and pStat3(−) cases and a Student's *t*-test *P*<0.05). Of the 214 differentially expressed genes, 150 genes were over-expressed and 64 genes were under-expressed in pStat3(+) cases relative to pStat3(−) cases.
(PDF)

Table S2 **Differential gene expression in primary breast cancer as a function of pStat3.** A microarray statistical analysis of 54 pStat3− versus 45 pStat3+ tumor specimens resulted in identifying 136 genes which were differentially expressed according to pStat3 status (at least 1.5 fold between the means of pStat3(+) and pStat3(−) cases and a Student's t-test P<0.05). Of the 136 differentially expressed genes, 115 were over-expressed and 21 were under-expressed in pStat3(+) cases relative to pStat3(−) cases.
(PDF)

Table S3 **Differential gene expression in breast cancer as a function of pStat3.** A comparison of differentially expressed genes in ER− breast cancer (214 genes) versus all (ER− and ER+) breast cancers (136 genes) as a function of pStat3 revealed 50 genes overlapping including *ENPP2*.
(PDF)
