# Supplementary figures and images for: Stat3 Mediates Expression of Autotaxin in Breast Cancer
Source: PLoS One. 2011 Nov 28;6(11):e27851. doi: 10.1371/journal.pone.0027851 (PMC3225372; doi:10.1371/journal.pone.0027851)

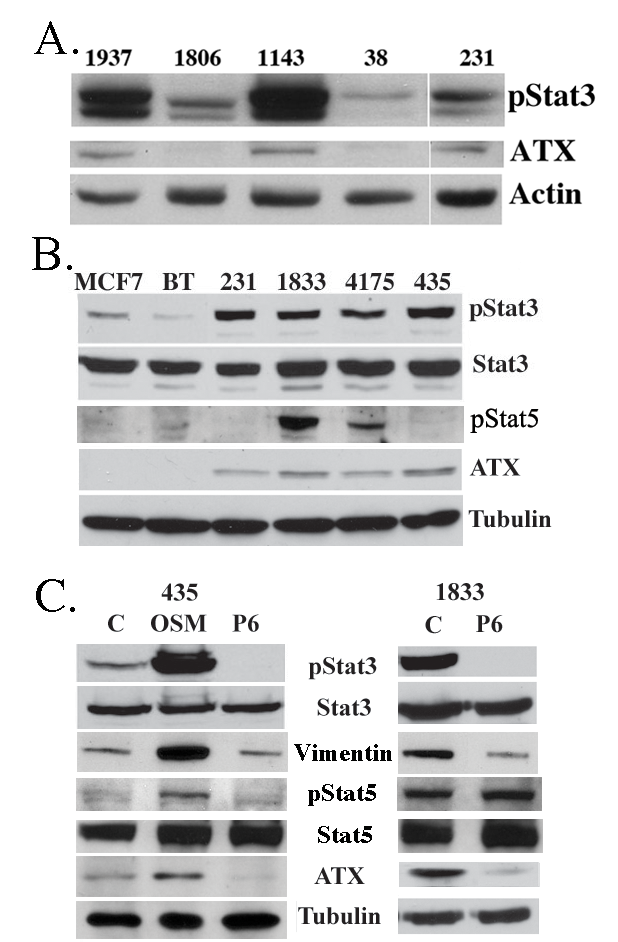

Supplement: Figure S1 — Stat3 regulation of ATX expression in triple negative breast cancer cells. A. Extracts (20 µg) isolated from 1937, 1806, 1143, 38 and MD-MB-231 cell lineswere analyzed for pStat3, ATX and Actin by western blot analysis. B. Extracts (20 µg) isolated from MCF7, BT474 (BT), MDA-MB-231 (231), 1833, 4175 and MDA-MB-435 (435) were analyzed for pStat3, Stat3, pStat5, ATX and Tubulin by western blot analysis. C. Whole cell extracts (50 (µg) isolated from MDA-MB-435 cells treated for 4 hours with dimethyl sulfoxide (C), OSM (5 ng/ml) and P6 (1 µM); 1833 cells treated with DMSO or P6 were analyzed for pStat3, Stat3, Vimentin, pStat5, Stat5, ATX and Tubulin by western blot analysis. (TIF) [file pone.0027851.s001.tif]
