# Supplementary material for: Stat3 Mediates Expression of Autotaxin in Breast Cancer
Source: PLoS One. 2011 Nov 28;6(11):e27851. doi: 10.1371/journal.pone.0027851 (PMC3225372; doi:10.1371/journal.pone.0027851)
Supplement: Table S1 — Differential gene expression in primary ER− breast cancer as a function of pStat3. A microarray statistical analysis of the 8 pStat3+ versus the 13 pStat3− tumor specimens identified 214 differentially expressed genes according to pStat3 status (at least two-fold between the means of pStat3(+) and pStat3(−) cases and a Student's t-test P<0.05). Of the 214 differentially expressed genes, 150 genes were over-expressed and 64 genes were under-expressed in pStat3(+) cases relative to pStat3(−) cases. (PDF) [file pone.0027851.s002.pdf]

# Supplemental Table1

| Probeset ID | Gene Symbol | p-value | Fold-Change | Probeset ID | Gene Symbol | p-value | Fold-Change |
|---|---|---|---|---|---|---|---|
| 209392_at | **ENPP2** | 4.2E-07 | 8.70 | 202973_x_at | FAM13A | 2.4E-04 | 2.51 |
| 210839_s_at | **ENPP2** | 2.0E-07 | 8.15 | 212298_at | NRP1 | 4.5E-05 | 2.51 |
| 206488_s_at | CD36 | 2.3E-03 | 4.01 | 203131_at | PDGFRA | 6.1E-03 | 2.50 |
| 218002_s_at | CXCL14 | 8.5E-03 | 3.73 | 202007_at | NID1 | 1.1E-03 | 2.50 |
| 204438_at | MRC1 / | 6.4E-03 | 3.54 | 201360_at | CST3 | 2.3E-04 | 2.49 |
| 213139_at | SNAI2 | 8.7E-03 | 3.39 | 208892_s_at | DUSP6 | 2.1E-02 | 2.47 |
| 206042_x_at | SNRPN / | 5.8E-05 | 3.31 | 204174_at | ALOX5AP | 1.7E-02 | 2.47 |
| 202409_at | IGF2 | 3.5E-02 | 3.26 | 211896_s_at | DCN | 2.9E-02 | 2.44 |
| 212192_at | KCTD12 | 6.6E-04 | 3.14 | 213943_at | TWIST1 | 1.6E-03 | 2.44 |
| 204298_s_at | LOX | 5.2E-03 | 3.00 | 209348_s_at | MAF | 1.1E-06 | 2.43 |
| 202709_at | FMOD | 1.8E-04 | 2.99 | 204052_s_at | SFRP4 | 3.4E-02 | 2.43 |
| 212097_at | CAV1 | 3.4E-05 | 2.97 | 201278_at | DAB2 | 5.5E-04 | 2.43 |
| 201116_s_at | CPE | 2.4E-04 | 2.97 | 205542_at | STEAP1 | 1.8E-02 | 2.40 |
| 209555_s_at | CD36 | 1.6E-02 | 2.97 | 214500_at | H2AFY | 2.3E-04 | 2.40 |
| 209541_at | IGF1 | 7.3E-03 | 2.91 | 209656_s_at | TMEM47 | 4.8E-02 | 2.39 |
| 213566_at | RNASE6 | 3.0E-04 | 2.90 | 211571_s_at | VCAN | 1.1E-02 | 2.38 |
| 219607_s_at | MS4A4A | 1.7E-02 | 2.90 | 204472_at | GEM | 3.4E-03 | 2.37 |
| 215446_s_at | LOX | 2.6E-03 | 2.88 | 201743_at | CD14 | 2.6E-02 | 2.36 |
| 219935_at | ADAMTS | 4.3E-03 | 2.88 | 211734_s_at | FCER1A | 1.1E-02 | 2.36 |
| 201522_x_at | SNRPN / | 3.2E-04 | 2.87 | 212865_s_at | COL14A1 | 2.5E-02 | 2.36 |
| 204955_at | SRPX | 5.5E-03 | 2.85 | 205624_at | CPA3 | 6.4E-03 | 2.35 |
| 218625_at | NRN1 | 1.3E-02 | 2.84 | 207977_s_at | DPT | 4.5E-02 | 2.35 |
| 209612_s_at | ADH1B | 3.0E-02 | 2.83 | 219049_at | CSGALNA | 1.5E-03 | 2.35 |
| 206584_at | LY96 | 3.3E-03 | 2.82 | 202207_at | ARL4C | 1.9E-03 | 2.33 |
| 201645_at | TNC | 1.8E-02 | 2.81 | 203561_at | FCGR2A | 2.2E-02 | 2.32 |
| 203065_s_at | CAV1 | 1.8E-04 | 2.81 | 201280_s_at | DAB2 | 3.4E-03 | 2.31 |
| 205943_at | TDO2 | 1.7E-02 | 2.78 | 201008_s_at | TXNIP | 8.6E-03 | 2.31 |
| 204774_at | EVI2A | 2.1E-04 | 2.76 | 202206_at | ARL4C | 4.7E-03 | 2.31 |
| 203305_at | F13A1 | 1.4E-02 | 2.74 | 218656_s_at | LHFP | 8.5E-04 | 2.30 |
| 213125_at | OLFML2 | 4.6E-03 | 2.67 | 213524_s_at | G0S2 | 2.4E-02 | 2.28 |
| 208944_at | TGFBR2 | 1.3E-04 | 2.66 | 201540_at | FHL1 | 6.0E-03 | 2.27 |
| 203548_s_at | LPL | 1.6E-02 | 2.64 | 219014_at | PLAC8 | 1.3E-02 | 2.27 |
| 201117_s_at | CPE | 4.4E-04 | 2.61 | 201505_at | LAMB1 | 2.3E-02 | 2.27 |
| 221796_at | NTRK2 | 4.6E-02 | 2.61 | 204959_at | MNDA | 2.8E-02 | 2.27 |
| 208131_s_at | PTGIS | 4.1E-03 | 2.61 | 204646_at | DPYD | 1.5E-03 | 2.26 |
| 202016_at | MEST | 9.7E-03 | 2.59 | 211506_s_at | IL8 | 1.6E-02 | 2.26 |
| 201279_s_at | DAB2 | 2.0E-04 | 2.57 | 205991_s_at | PRRX1 | 5.4E-03 | 2.26 |
| 213258_at | TFPI | 1.2E-04 | 2.57 | 202741_at | PRKACB | 3.4E-04 | 2.25 |
| 203504_s_at | ABCA1 | 3.4E-05 | 2.54 | 209200_at | MEF2C | 5.4E-04 | 2.25 |
| 202746_at | ITM2A | 1.3E-02 | 2.54 | 209047_at | AQP1 /// | 9.7E-04 | 2.24 |
| 201667_at | GJA1 | 1.5E-02 | 2.53 | 204122_at | TYROBP | 1.8E-02 | 2.24 |
| 215646_s_at | VCAN | 8.3E-03 | 2.53 | 204688_at | SGCE | 2.7E-03 | 2.24 |
| 209613_s_at | ADH1B | 3.2E-02 | 2.53 | 202113_s_at | SNX2 | 3.9E-03 | 2.24 |
| 207808_s_at | PROS1 | 1.5E-03 | 2.53 | 205226_at | PDGFRL | 4.2E-03 | 2.24 |
| 218718_at | PDGFC | 8.1E-04 | 2.53 | 210764_s_at | CYR61 | 3.0E-03 | 2.22 |
| 215388_s_at | CFH / | 3.0E-04 | 2.53 | 204563_at | SELL | 1.7E-02 | 2.21 |
| 212588_at | PTPRC | 1.0E-02 | 2.52 | 202878_s_at | CD93 | 1.8E-03 | 2.20 |
| 211742_s_at | EVI2B | 1.3E-03 | 2.52 | 202202_s_at | LAMA4 | 6.0E-03 | 2.20 |

| Probeset ID | Gene Symbol | p-value | Fold-Change | Probeset ID | Gene Symbol | p-value | Fold-Change |
|---|---|---|---|---|---|---|---|
| 206332_s_at | IFI16 | 1.2E-02 | 2.20 | 219947_at | CLEC4A | 2.9E-02 | 2.03 |
| 210757_x_at | DAB2 | 7.9E-04 | 2.20 | 212587_s_at | PTPRC | 5.2E-03 | 2.02 |
| 221210_s_at | NPL | 1.5E-02 | 2.19 | 203562_at | FEZ1 | 1.3E-04 | 2.02 |
| 210946_at | PPAP2A | 4.9E-03 | 2.18 | 201581_at | TMX4 | 1.0E-03 | 2.02 |
| 218589_at | LPAR6 | 1.0E-03 | 2.18 | 205683_x_at | TPSAB1 | 4.9E-02 | 2.01 |
| 203104_at | CSF1R | 9.1E-04 | 2.18 | 207134_x_at | TPSB2 | 3.2E-02 | 2.00 |
| 209540_at | IGF1 | 6.8E-03 | 2.17 | 214290_s_at | HIST2H2A | 2.9E-02 | -2.00 |
| 208891_at | DUSP6 | 3.7E-02 | 2.17 | 213050_at | COBL | 2.2E-02 | -2.01 |
| 217047_s_at | FAM13A | 9.4E-05 | 2.17 | 202870_s_at | CDC20 | 3.9E-03 | -2.02 |
| 211813_x_at | DCN | 4.1E-02 | 2.16 | 209529_at | PPAP2C | 3.8E-02 | -2.02 |
| 222108_at | AMIGO2 | 1.6E-02 | 2.16 | 201292_at | TOP2A | 5.9E-03 | -2.03 |
| 209147_s_at | PPAP2A | 4.8E-03 | 2.15 | 204822_at | TTK | 7.0E-03 | -2.04 |
| 219759_at | ERAP2 | 2.4E-02 | 2.15 | 209260_at | SFN | 2.1E-03 | -2.04 |
| 207238_s_at | PTPRC | 3.9E-03 | 2.14 | 204654_s_at | TFAP2A | 1.8E-02 | -2.04 |
| 208983_s_at | PECAM1 | 4.9E-03 | 2.14 | 210559_s_at | CDC2 | 8.0E-04 | -2.05 |
| 221558_s_at | LEF1 | 3.0E-03 | 2.14 | 204026_s_at | ZWINT | 7.0E-06 | -2.07 |
| 200920_s_at | BTG1 | 4.0E-03 | 2.12 | 204679_at | KCNK1 | 1.6E-02 | -2.07 |
| 210889_s_at | FCGR2B | 2.5E-02 | 2.12 | 202575_at | CRABP2 | 2.6E-02 | -2.08 |
| 201720_s_at | LAPTM5 | 1.2E-02 | 2.12 | 203213_at | CDC2 | 9.1E-07 | -2.10 |
| 203887_s_at | THBD | 8.3E-03 | 2.11 | 208779_x_at | DDR1 | 2.0E-02 | -2.11 |
| 202948_at | IL1R1 | 3.2E-02 | 2.11 | 202708_s_at | HIST2H2B | 3.6E-02 | -2.11 |
| 213416_at | ITGA4 | 2.5E-05 | 2.10 | 202954_at | UBE2C | 4.9E-04 | -2.12 |
| 205898_at | CX3CR1 | 3.6E-04 | 2.10 | 218039_at | NUSAP1 | 4.5E-04 | -2.13 |
| 217889_s_at | CYBRD1 | 3.9E-03 | 2.09 | 202705_at | CCNB2 | 5.4E-06 | -2.13 |
| 208893_s_at | DUSP6 | 1.3E-02 | 2.09 | 204170_s_at | CKS2 | 1.1E-02 | -2.13 |
| 209581_at | PLA2G1 | 3.8E-02 | 2.09 | 219121_s_at | ESRP1 | 5.1E-03 | -2.15 |
| 201109_s_at | THBS1 | 1.2E-02 | 2.09 | 208079_s_at | AURKA | 4.8E-05 | -2.17 |
| 204150_at | STAB1 | 5.1E-03 | 2.09 | 209008_x_at | KRT8 | 4.0E-02 | -2.19 |
| 211671_s_at | NR3C1 | 7.2E-04 | 2.09 | 219959_at | MOCOS | 1.1E-02 | -2.21 |
| 200762_at | DPYSL2 | 6.0E-03 | 2.08 | 203876_s_at | MMP11 | 1.2E-03 | -2.22 |
| 208966_x_at | IFI16 | 2.5E-02 | 2.08 | 203744_at | HMGB3 | 5.4E-04 | -2.22 |
| 208965_s_at | IFI16 | 9.4E-03 | 2.08 | 218542_at | CEP55 | 2.6E-06 | -2.22 |
| 204115_at | GNG11 | 6.3E-03 | 2.08 | 205807_s_at | TUFT1 | 7.7E-03 | -2.22 |
| 209199_s_at | MEF2C | 3.1E-04 | 2.07 | 214240_at | GAL | 2.2E-02 | -2.26 |
| 207173_x_at | CDH11 | 1.8E-02 | 2.07 | 202580_x_at | FOXM1 | 1.6E-03 | -2.27 |
| 219872_at | C4orf18 | 2.0E-03 | 2.06 | 218186_at | RAB25 | 3.7E-02 | -2.28 |
| 211651_s_at | LAMB1 | 1.3E-02 | 2.06 | 205428_s_at | CALB2 | 1.6E-02 | -2.31 |
| 220330_s_at | SAMSN1 | 5.8E-03 | 2.06 | 212070_at | GPR56 | 1.5E-02 | -2.32 |
| 201865_x_at | NR3C1 | 3.6E-04 | 2.06 | 202525_at | PRSS8 | 1.5E-02 | -2.32 |
| 204638_at | ACP5 | 3.6E-02 | 2.06 | 214469_at | HIST1H2A | 9.8E-03 | -2.34 |
| 209732_at | CLEC2B | 1.9E-03 | 2.06 | 221854_at | PKP1 | 2.4E-02 | -2.35 |
| 212764_at | ZEB1 | 1.6E-03 | 2.05 | 204127_at | RFC3 | 2.3E-03 | -2.38 |
| 211676_s_at | IFNGR1 | 8.6E-04 | 2.05 | 37117_at | ARHGAP8 | 1.1E-02 | -2.38 |
| 204834_at | FGL2 | 1.9E-02 | 2.05 | 204641_at | NEK2 | 8.7E-05 | -2.39 |
| 204220_at | GMFG | 3.0E-03 | 2.04 | 207828_s_at | CENPF | 3.8E-04 | -2.42 |
| 203799_at | CD302 | 7.1E-03 | 2.04 | 213906_at | MYBL1 | 4.1E-02 | -2.42 |
| 204057_at | IRF8 | 1.0E-02 | 2.04 | 203407_at | PPL | 7.3E-03 | -2.45 |
| 219666_at | MS4A6A | 3.1E-02 | 2.03 | 203407_at | PPL | 7.3E-03 | -2.45 |

| Probeset ID | Gene Symbol | p-value | Fold-Change |
|---|---|---|---|
| 208190_s_at | LSR | 3.1E-02 | -2.47 |
| 203878_s_at | MMP11 | 2.4E-03 | -2.56 |
| 212531_at | LCN2 | 3.3E-02 | -2.72 |
| 33322_i_at | SFN | 1.6E-03 | -2.74 |
| 203108_at | GPRC5A | 1.3E-02 | -2.75 |
| 204086_at | PRAME | 1.7E-02 | -2.78 |
| 33323_r_at | SFN | 1.9E-03 | -2.80 |
| 220196_at | MUC16 | 2.7E-02 | -2.87 |
| 202581_at | HSPA1A | 8.1E-03 | -2.98 |
| 205350_at | CRABP1 | 1.1E-02 | -3.00 |
| 200606_at | DSP | 9.1E-03 | -3.14 |
| 208651_x_at | CD24 | 2.0E-02 | -3.17 |
| 209771_x_at | CD24 | 2.1E-02 | -3.69 |
| 266_s_at | CD24 | 2.2E-02 | -3.72 |
| 204885_s_at | MSLN | 3.0E-02 | -3.79 |
| 201131_s_at | CDH1 | 8.9E-03 | -3.88 |
| 215729_s_at | VGLL1 | 2.6E-03 | -4.09 |
| 216379_x_at | CD24 | 1.5E-02 | -4.14 |
| 208650_s_at | CD24 | 2.3E-02 | -4.26 |
| 205916_at | S100A7 | 1.8E-02 | -4.33 |
| 201650_at | KRT19 | 6.4E-03 | -6.03 |
