# Supplementary material for: Stat3 Mediates Expression of Autotaxin in Breast Cancer
Source: PLoS One. 2011 Nov 28;6(11):e27851. doi: 10.1371/journal.pone.0027851 (PMC3225372; doi:10.1371/journal.pone.0027851)
Supplement: Table S2 — Differential gene expression in primary breast cancer as a function of pStat3. A microarray statistical analysis of 54 pStat3− versus 45 pStat3+ tumor specimens resulted in identifying 136 genes which were differentially expressed according to pStat3 status (at least 1.5 fold between the means of pStat3(+) and pStat3(−) cases and a Student's t-test P<0.05). Of the 136 differentially expressed genes, 115 were over-expressed and 21 were under-expressed in pStat3(+) cases relative to pStat3(−) cases. (PDF) [file pone.0027851.s003.pdf]

Supplemental Table 2

Probeset ID	Gene Symbol	p-value	Fold-Change	Probeset ID	Gene Symbol	p-value	Fold-Change
203980_at	FABP4	8.0E-04	2.35	221841_s_at	KLF4	1.9E-03	1.60
218002_s_at	CXCL14	4.6E-03	2.33	201952_at	ALCAM	1.3E-02	1.60
214451_at	TFAP2B	4.5E-03	2.28	204070_at	RARRES3	2.0E-02	1.60
201525_at	APOD	6.5E-03	2.17	217528_at	CLCA2	1.3E-02	1.60
209392_at	**ENPP2**	1.3E-06	2.14	209505_at	NR2F1	7.1E-05	1.59
210839_s_at	**ENPP2**	4.3E-07	2.03	205542_at	STEAP1	2.0E-03	1.59
204472_at	GEM	1.0E-06	1.99	205624_at	CPA3	3.4E-04	1.59
209541_at	IGF1	7.7E-06	1.95	210946_at	PPAP2A	2.0E-07	1.59
218313_s_at	GALNT7	6.6E-04	1.94	203789_s_at	SEMA3C	1.7E-02	1.59
212192_at	KCTD12	2.7E-06	1.90	209147_s_at	PPAP2A	6.5E-07	1.59
202437_s_at	CYP1B1	9.2E-04	1.86	201041_s_at	DUSP1	4.8E-04	1.58
204041_at	MAOB	9.0E-04	1.85	214774_x_at	TOX3	2.1E-02	1.58
204014_at	DUSP4	1.6E-02	1.85	207808_s_at	PROS1	3.3E-05	1.58
212865_s_at	COL14A	1.7E-05	1.84	200762_at	DPYSL2	6.2E-05	1.58
202746_at	ITM2A	1.6E-05	1.83	216623_x_at	TOX3	1.6E-02	1.58
206488_s_at	CD36	2.6E-04	1.81	209613_s_at	ADH1B	1.7E-03	1.58
208892_s_at	DUSP6	1.4E-04	1.80	202748_at	GBP2	3.8E-04	1.58
202687_s_at	TNFSF1	2.7E-03	1.80	203131_at	PDGFRA	1.5E-03	1.58
210896_s_at	ASPH	3.1E-05	1.78	209555_s_at	CD36	8.0E-03	1.57
204455_at	DST	3.3E-03	1.78	209335_at	DCN	2.8E-03	1.57
200795_at	SPARCL	3.3E-05	1.77	202291_s_at	MGP	3.3E-02	1.57
212224_at	ALDH1A	2.2E-06	1.77	202478_at	TRIB2	3.0E-06	1.57
213258_at	TFPI	1.6E-06	1.76	206584_at	LY96	7.0E-04	1.57
218541_s_at	C8orf4	1.4E-02	1.75	203710_at	ITPR1	6.8E-04	1.56
206165_s_at	CLCA2	3.3E-03	1.74	204897_at	PTGER4	9.3E-05	1.56
208891_at	DUSP6	3.3E-04	1.73	208131_s_at	PTGIS	3.0E-04	1.56
202688_at	TNFSF1	5.5E-03	1.73	205382_s_at	CFD	1.6E-02	1.56
201427_s_at	SEPP1	8.4E-04	1.73	216321_s_at	NR3C1	3.7E-05	1.56
217889_s_at	CYBRD1	7.8E-04	1.72	208763_s_at	TSC22D3	6.9E-04	1.55
209612_s_at	ADH1B	1.6E-03	1.72	222108_at	AMIGO2	3.7E-02	1.55
201645_at	TNC	1.2E-03	1.72	201311_s_at	SH3BGRL	6.7E-04	1.55
214428_x_at	C4A	1.3E-02	1.71	202609_at	EPS8	8.1E-05	1.55
212196_at	IL6ST	1.7E-03	1.71	213397_x_at	RNASE4	7.8E-04	1.55
209135_at	ASPH	7.2E-05	1.70	201445_at	CNN3	7.4E-04	1.55
208451_s_at	C4A	2.9E-02	1.68	221760_at	MAN1A1	1.6E-03	1.54
212195_at	IL6ST	6.1E-03	1.67	204036_at	LPAR1	3.7E-06	1.54
200670_at	XBP1	1.3E-02	1.67	209047_at	AQP1	1.5E-04	1.54
211896_s_at	DCN	2.3E-03	1.66	204580_at	MMP12	4.2E-02	1.53
208335_s_at	DARC	9.0E-06	1.66	204438_at	MRC1MRC	4.8E-03	1.53
208893_s_at	DUSP6	3.0E-05	1.65	212298_at	NRP1	1.2E-05	1.53
209687_at	CXCL12	6.4E-03	1.65	201694_s_at	EGR1	1.4E-03	1.53
218656_s_at	LHFP	2.5E-06	1.64	209189_at	FOS	8.2E-03	1.53
204051_s_at	SFRP4	6.2E-03	1.63	221024_s_at	SLC2A10	2.3E-02	1.53
209894_at	LEPR	7.8E-07	1.63	213519_s_at	LAMA2	6.3E-05	1.53
202436_s_at	CYP1B1	1.2E-03	1.63	201012_at	ANXA1	5.4E-03	1.53
205158_at	RNASE4	2.9E-04	1.63	201540_at	FHL1	2.6E-04	1.53
211737_x_at	PTN	7.6E-03	1.63	201941_at	CPD	4.3E-04	1.53
218353_at	RGS5	9.7E-03	1.62	204112_s_at	HNMT	9.4E-05	1.52
212956_at	TBC1D9	4.8E-02	1.62	221748_s_at	TNS1	5.0E-05	1.52
204646_at	DPYD	9.8E-07	1.61	214895_s_at	ADAM10	3.0E-05	1.52

208944_at	TGFBR2	7.7E-06	1.60
202016_at	MEST	3.1E-03	1.60
221841_s_at	KLF4	1.9E-03	1.60
201952_at	ALCAM	1.3E-02	1.60
204070_at	RARRES	2.0E-02	1.60
217528_at	CLCA2	1.3E-02	1.60
209505_at	NR2F1	7.1E-05	1.59
205542_at	STEAP1	2.0E-03	1.59
205624_at	CPA3	3.4E-04	1.59
210946_at	PPAP2A	2.0E-07	1.59
203789_s_at	SEMA3C	1.7E-02	1.59
209147_s_at	PPAP2A	6.5E-07	1.59
201041_s_at	DUSP1	4.8E-04	1.58
214774_x_at	TOX3	2.1E-02	1.58
207808_s_at	PROS1	3.3E-05	1.58
200762_at	DPYSL2	6.2E-05	1.58
216623_x_at	TOX3	1.6E-02	1.58
209613_s_at	ADH1B	1.7E-03	1.58
202748_at	GBP2	3.8E-04	1.58
203131_at	PDGFRA	1.5E-03	1.58
209555_s_at	CD36	8.0E-03	1.57
209335_at	DCN	2.8E-03	1.57
202291_s_at	MGP	3.3E-02	1.57
202478_at	TRIB2	3.0E-06	1.57
206584_at	LY96	7.0E-04	1.57
203710_at	ITPR1	6.8E-04	1.56
204897_at	PTGER4	9.3E-05	1.56
208131_s_at	PTGIS	3.0E-04	1.56
205382_s_at	CFD	1.6E-02	1.56
216321_s_at	NR3C1	3.7E-05	1.56
208763_s_at	TSC22D	6.9E-04	1.55
222108_at	AMIGO2	3.7E-02	1.55
201311_s_at	SH3BGR	6.7E-04	1.55
202609_at	EPS8	8.1E-05	1.55
213397_x_at	RNASE4	7.8E-04	1.55
201445_at	CNN3	7.4E-04	1.55
221760_at	MAN1A1	1.6E-03	1.54
204036_at	LPAR1	3.7E-06	1.54
209047_at	AQP1 ///	1.5E-04	1.54
204580_at	MMP12	4.2E-02	1.53
204438_at	MRC1 ///	4.8E-03	1.53
212298_at	NRP1	1.2E-05	1.53
201694_s_at	EGR1	1.4E-03	1.53
209189_at	FOS	8.2E-03	1.53
221024_s_at	SLC2A10	2.3E-02	1.53
213519_s_at	LAMA2	6.3E-05	1.53
201012_at	ANXA1	5.4E-03	1.53
201540_at	FHL1	2.6E-04	1.53
201941_at	CPD	4.3E-04	1.53
204112_s_at	HNMT	9.4E-05	1.52
221748_s_at	TNS1	5.0E-05	1.52
214895_s_at	ADAM10	3.0E-05	1.52
34210_at	CD52	1.1E-02	1.51
215388_s_at	CFH ///	7.7E-06	1.51
34210_at	CD52	1.1E-02	1.51
218736_s_at	PALMD	1.1E-04	1.51
201842_s_at	EFEMP1	2.1E-02	1.51
201466_s_at	JUN	1.9E-04	1.51
202628_s_at	SERPIN	2.1E-03	1.51
201312_s_at	SH3BGR	3.7E-03	1.51
202731_at	PDCD4	1.4E-02	1.51
218804_at	ANO1	1.7E-02	1.51
201117_s_at	CPE	5.0E-04	1.51
201943_s_at	CPD	2.8E-04	1.50
201581_at	TMX4	7.1E-06	1.50
202113_s_at	SNX2	5.9E-05	1.50
214290_s_at	HIST2H2	2.5E-02	-1.50
210387_at	HIST1H2	2.8E-02	-1.52
205358_at	GRIA2	4.1E-02	-1.54
202870_s_at	CDC20	1.6E-03	-1.54
215729_s_at	VGLL1	3.2E-02	-1.56
201195_s_at	SLC7A5	5.5E-03	-1.56
205334_at	S100A1	2.0E-02	-1.58
206074_s_at	HMGA1	1.6E-04	-1.58
209125_at	KRT6A	1.4E-02	-1.58
220624_s_at	ELF5	2.5E-03	-1.58
203628_at	IGF1R	1.3E-02	-1.59
214469_at	HIST1H2	6.8E-04	-1.60
204654_s_at	TFAP2A	2.6E-04	-1.61
214472_at	HIST1H	2.2E-04	-1.64
202975_s_at	RHOBTB	6.7E-04	-1.65
213906_at	MYBL1	1.1E-02	-1.66
205350_at	CRABP1	8.1E-03	-1.79
220625_s_at	ELF5	7.7E-03	-1.82
204885_s_at	MSLN	1.3E-03	-1.83
220559_at	EN1	1.3E-02	-1.87
201131_s_at	CDH1	5.3E-04	-2.16
