# Supplementary material for: Stat3 Mediates Expression of Autotaxin in Breast Cancer
Source: PLoS One. 2011 Nov 28;6(11):e27851. doi: 10.1371/journal.pone.0027851 (PMC3225372; doi:10.1371/journal.pone.0027851)
Supplement: Table S3 — Differential gene expression in breast cancer as a function of pStat3. A comparison of differentially expressed genes in ER− breast cancer (214 genes) versus all (ER− and ER+) breast cancers (136 genes) as a function of pStat3 revealed 50 genes overlapping including ENPP2. (PDF) [file pone.0027851.s004.pdf]

Supplemental Table 3

Probeset ID	Gene Symbol	p-value	Fold-Change	Gene Symbol	p-value	Fold-Change
218002_s_at	CXCL14	4.7E-03	2.33	CXCL14	8.5E-03	3.73
209392_at	ENPP2	1.3E-06	2.14	ENPP2	4.2E-07	8.70
210839_s_at	ENPP2	4.3E-07	2.03	ENPP2	2.0E-07	8.15
204472_at	GEM	1.0E-06	1.99	GEM	3.4E-03	2.37
209541_at	IGF1	7.7E-06	1.95	IGF1	7.3E-03	2.91
212192_at	KCTD12	2.7E-06	1.90	KCTD12	6.6E-04	3.14
212865_s_at	COL14A1	1.7E-05	1.84	COL14A1	2.5E-02	2.36
202746_at	ITM2A	1.6E-05	1.83	ITM2A	1.3E-02	2.54
206488_s_at	CD36	2.6E-04	1.81	CD36	2.3E-03	4.01
208892_s_at	DUSP6	1.4E-04	1.80	DUSP6	2.1E-02	2.47
213258_at	TFPI	1.6E-06	1.76	TFPI	1.2E-04	2.57
208891_at	DUSP6	3.3E-04	1.73	DUSP6	3.7E-02	2.17
217889_s_at	CYBRD1	7.8E-04	1.72	CYBRD1	3.9E-03	2.09
209612_s_at	ADH1B	1.6E-03	1.72	ADH1B	3.0E-02	2.83
201645_at	TNC	1.2E-03	1.72	TNC	1.8E-02	2.81
211896_s_at	DCN	2.3E-03	1.66	DCN	2.9E-02	2.44
208893_s_at	DUSP6	3.0E-05	1.65	DUSP6	1.3E-02	2.09
218656_s_at	LHFP	2.5E-06	1.64	LHFP	8.5E-04	2.30
204646_at	DPYD	9.8E-07	1.61	DPYD	1.5E-03	2.26
208944_at	TGFBR2	7.7E-06	1.60	TGFBR2	1.3E-04	2.66
202016_at	MEST	3.1E-03	1.60	MEST	9.7E-03	2.59
205542_at	STEAP1	2.0E-03	1.59	STEAP1	1.8E-02	2.40
205624_at	CPA3	3.4E-04	1.59	CPA3	6.4E-03	2.35
210946_at	PPAP2A	2.0E-07	1.59	PPAP2A	4.9E-03	2.18
209147_s_at	PPAP2A	6.5E-07	1.59	PPAP2A	4.8E-03	2.15
207808_s_at	PROS1	3.3E-05	1.58	PROS1	1.5E-03	2.53
200762_at	DPYSL2	6.2E-05	1.58	DPYSL2	6.0E-03	2.08
209613_s_at	ADH1B	1.7E-03	1.58	ADH1B	3.2E-02	2.53
203131_at	PDGFRA	1.5E-03	1.58	PDGFRA	6.1E-03	2.50
209555_s_at	CD36	8.0E-03	1.57	CD36	1.6E-02	2.97
206584_at	LY96	7.0E-04	1.57	LY96	3.3E-03	2.82
208131_s_at	PTGIS	3.0E-04	1.56	PTGIS	4.1E-03	2.61
222108_at	AMIGO2	3.7E-02	1.55	AMIGO2	1.6E-02	2.16
209047_at	AQP1	1.5E-04	1.54	AQP1	9.7E-04	2.24
204438_at	MRC1	4.8E-03	1.53	MRC1	6.4E-03	3.54
212298_at	NRP1	1.2E-05	1.53	NRP1	4.5E-05	2.51
201540_at	FHL1	2.6E-04	1.53	FHL1	6.0E-03	2.27
215388_s_at	CFH	7.7E-06	1.51	CFH	3.0E-04	2.53
201117_s_at	CPE	5.0E-04	1.51	CPE	4.4E-04	2.61
201581_at	TMX4	7.1E-06	1.50	TMX4	1.0E-03	2.02
202113_s_at	SNX2	5.9E-05	1.50	SNX2	3.9E-03	2.24
214290_s_at	HIST2H2AA3	2.5E-02	-1.50	HIST2H2AA3	2.9E-02	-2.00
202870_s_at	CDC20	1.6E-03	-1.54	CDC20	3.9E-03	-2.02
215729_s_at	VGLL1	3.2E-02	-1.56	VGLL1	2.6E-03	-4.09
214469_at	HIST1H2AE	6.8E-04	-1.60	HIST1H2AE	9.8E-03	-2.34
204654_s_at	TFAP2A	2.6E-04	-1.61	TFAP2A	1.8E-02	-2.04
213906_at	MYBL1	1.1E-02	-1.66	MYBL1	4.1E-02	-2.42
205350_at	CRABP1	8.1E-03	-1.79	CRABP1	1.1E-02	-3.00
204885_s_at	MSLN	1.3E-03	-1.83	MSLN	3.0E-02	-3.79
201131_s_at	CDH1	5.3E-04	-2.16	CDH1	8.9E-03	-3.88
